# Protozoan Parasite *Babesia microti* Subverts Adaptive Immunity and Enhances Lyme Disease Severity

**DOI:** 10.3389/fmicb.2019.01596

**Published:** 2019-07-10

**Authors:** Vitomir Djokic, Lavoisier Akoolo, Shekerah Primus, Samantha Schlachter, Kathleen Kelly, Purnima Bhanot, Nikhat Parveen

**Affiliations:** ^1^Department of Microbiology, Biochemistry and Molecular Genetics, Rutgers New Jersey Medical School, Newark, NJ, United States; ^2^Biomedical Sciences, Cornell University College of Veterinary Medicine, Ithaca, NY, United States

**Keywords:** *Borrelia burgdorferi*, *Babesia microti*, Lyme disease, babesiosis, co-infection, tick-borne co-infection, adaptive immune response

## Abstract

Lyme disease is the most prominent tick-borne disease in the United States. Co-infections with the tick-transmitted pathogens *Babesia microti* and *Borrelia burgdorferi* sensu stricto are becoming a serious health problem. *B. burgdorferi* is an extracellular spirochete that causes Lyme disease while *B. microti* is a protozoan that infects erythrocytes and causes babesiosis. Testing of donated blood for *Babesia* species is not currently mandatory due to unavailability of an FDA approved test. Transmission of this protozoan by blood transfusion often results in high morbidity and mortality in recipients. Infection of C3H/HeJ mice with *B. burgdorferi* and *B. microti* individually results in inflammatory Lyme disease and display of human babesiosis-like symptoms, respectively. Here we use this mouse model to provide a detailed investigation of the reciprocal influence of the two pathogens on each other during co-infection. We show that *B. burgdorferi* infection attenuates parasitemia in mice while *B. microti* subverts the splenic immune response, such that a marked decrease in splenic B and T cells, reduction in antibody levels and diminished functional humoral immunity, as determined by spirochete opsonophagocytosis, are observed in co-infected mice compared to only *B. burgdorferi* infected mice. Furthermore, immunosuppression by *B. microti* in co-infected mice showed an association with enhanced Lyme disease manifestations. This study demonstrates the effect of only simultaneous infection by *B. burgdorferi* and *B. microti* on each pathogen, immune response and on disease manifestations with respect to infection by the spirochete and the parasite. In our future studies, we will examine the overall effects of sequential infection by these pathogens on host immune responses and disease outcomes.

## Introduction

The Centers of Disease Control and Prevention (CDC) estimates that ∼300,000 cases of Lyme disease and ∼2000 cases of human babesiosis occur in the United States annually, while ∼65,000 cases of Lyme disease are reported occur in Europe per year (Moore et al., 2016; Primus et al., 2018). However, projected case number in Germany alone was >200,000 per year emphasizing under-reporting of Lyme disease also in Europe (Muller et al., 2012). The Lyme disease causing spirochete *Borrelia burgdorferi* is an extracellular bacterial pathogen that may invade the skin, musculoskeletal system, heart, joints and neuronal system. In the United States, Lyme arthritis is the most common persistent manifestation while acrodermatitis and severe neuroborreliosis are more common in Europe (Jungnick et al., 2015; Steere et al., 2016). The protozoan parasites, *Babesia microti* and *Babesia divergens* are the major causes of human babesiosis in the United States and Europe, respectively. Babesiosis is generally asymptomatic in healthy individuals, which often results in establishment of a carrier state, such that donation of blood by infected, asymptomatic individuals can lead to transfusion-transmitted babesiosis (Krause et al., 1998, 2008), making this disease a serious health concern.

Concurrent infection with protozoan parasites and various bacterial pathogens occurs frequently (Cox, 2001). *Ixodes* species tick populations have been increasing in the endemic regions and beyond and these vectors can transmit both *B. burgdorferi* and *B. microti* (Piesman et al., 1986; Jaenson et al., 2012; Lommano et al., 2012; Rizzoli et al., 2014; Johnson et al., 2017, 2018; Hahn et al., 2018; Piedmonte et al., 2018). The rise in incidence of *B. microti* and *B. burgdorferi* co-infections in humans appears to be driven primarily by increased co-infection of their common vector, ticks of the *Ixodes* species, which are capable of transmitting both pathogens simultaneously (Schulze et al., 2013; Dunn et al., 2014; Hersh et al., 2014; Knapp and Rice, 2015; Diuk-Wasser et al., 2016; Moutailler et al., 2016; Edwards et al., 2019). Although overall tick-borne co-infection rates are not yet documented in the United States, incidence of Lyme spirochetes and *B. microti* co-infections were as high as 40% in studies conducted with patient samples in two states in the Eastern United States, New Jersey and Connecticut (Diuk-Wasser et al., 2016; Primus et al., 2018). *B. microti-B. burgdorferi* co-infected patients suffer from significantly more diverse and intense symptoms that persist longer than in patients infected with *B. burgdorferi* alone (Krause et al., 1996). Severe disease often requires patient hospitalization, and can even cause death due to multi-organ failure (Martinez-Balzano et al., 2015), emphasizing the need for a comprehensive evaluation of the effect of co-infections using susceptible animal models.

Two previous co-infection studies performed in mice reported contradictory results regarding the effect of concomitant *B. microti* infection on the severity of Lyme disease (Moro et al., 2002; Coleman et al., 2005). Neither study provided insight into the effect of *B. burgdorferi* (s.s.) infection on babesiosis. Our study was undertaken to provide the first description of the reciprocal interaction of the two pathogens, *B. microti* and *B. burgdorferi* sensu stricto (referred as *B. burgdorferi* hereafter), and the impact of co-infections on pathogenic mechanisms of the two diseases. We selected young C3H/HeJ mice for our experiments because they exhibit Lyme arthritis and carditis similar to humans (Barthold et al., 1990; Armstrong et al., 1992), and also display *B. microti* parasitemia, splenomegaly and anemia (Moro et al., 2002; Coleman et al., 2005). Splenic cells of *B. burgdorferi* infected C3H mice showed an increase in B and CD4+ lymphocytes, increased IFN-γ production and diminished IL-4 levels (Keane-Myers and Nickell, 1995; Anguita et al., 1996; Kang et al., 1997; Zeidner et al., 1997; Glickstein et al., 2001) suggesting that in addition to the innate immune response, humoral immunity as well as Th1 and Th2 responses are important for spirochetes clearance. The innate immune response, involving macrophage and NK cells, is also important in controlling protozoan infections including *B. microti* (Aguilar-Delfin et al., 2001; Hunter and Sibley, 2012; Basso and Marini, 2014). In C57BL/6 mice, it is critical for conferring resistance to highly infectious WA-1 strain of *Babesia* species (Aguilar-Delfin et al., 2003). In this study, we investigated the impact of splenic immune responses on the resolution of *B. microti* parasitemia at the acute phase of co-infection with *B. burgdorferi*. We also assessed the effect of changes in the adaptive immune response caused by infection with *B. microti* on the clearance of Lyme spirochetes. Thus, we show the effect of modulation of splenic immune response by *B. microti* on the persistence and severity of Lyme disease manifestations in co-infected mice even after resolution of parasitemia in mice. Our studies provide tools and an animal model to investigate the effects of a past or active infection with an undetectable *B. microti* parasitemia on Lyme disease.

## Materials and Methods

### Animal Studies Ethics Statement

The Institutional Animal Care and Use Committee (IACUC) members reviewed and approved the protocol number PROTO201702491 entitled, “Spirochetes and tick-borne pathogens,” of the corresponding author to conduct this study at Rutgers New Jersey Medical School following guidelines of the Animal Welfare Act, The Institute of Laboratory Animal Resources Guide for the Care and Use of Laboratory Animals, and the Public Health Service Policy that are fully adopted at the Rutgers University.

### *B. burgdorferi* and *B. microti* Culture, Maintenance and Infection of Mice

**FIGURE 1:**
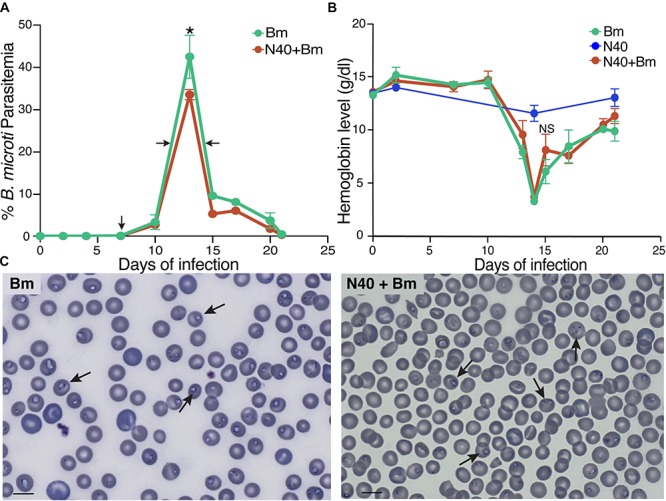
Effect of *B. burgdorferi* strain (N40) co-infection on *B. microti* (*Bm*) growth. **(A)** Percent of RBCs infected with *B. microti* in C3H mice infected either with *B. microti* alone, or with *B. microti* and N40, was determined over 21 days p.i. Each data point represents average parasitemia ± s.d. (*n* = 5). Both groups of mice exhibited peak parasitemia at 13th day p.i. with significantly high peak parasitemia in *Bm-*infected mice (^*^p < 0.05, *df* = 8, *F* = 1.72). **(B)** Hemoglobin levels were measured for 21 days p.i. N40-infected mice were used as controls. **(C)** Examination of blood smears showed lower erythrocytes density and higher parasitemia in *Bm-*infected mice (left) than co-infected mice (right). Bar represents 10 μm and arrows indicate parasitized RBCs.

Bioluminescent *B. burgdorferi* N40 strain was grown in BSKII medium containing 6% rabbit serum at 33^∘^C until logarithmic phase. *B. burgdorferi* numbers were then adjusted to 10^4^ spirochetes/ml of medium and 100 μl (10^3^ spirochetes) injected subcutaneously (sc) in each mouse (Chan et al., 2015). Disseminated infection followed by colonization of organs and tissues by bioluminescent N40 was monitored weekly by live imaging, as described previously (Chan et al., 2015) using IVIS-200 (Perkin-Elmer). *B. microti* (ATCC30221) was first inoculated in C3H/SCID female mice to obtain inoculum for subsequent experiments and parasitemia determined using Giemsa-stained thin blood smears according to CLSI guidelines (Garcia et al., 2000). C3H/HeJ mice for the experiments were purchased from Jackson Laboratory. Mice were infected for experimental purpose with 10^4^ *B. microti* infected RBCs as described previously (Djokic et al., 2018a, b). Female mice were used in all experiments to eliminate interference due to hormonal differences that sometimes affect parasitic infections. Young, 4-week-old mice were used because they display both Lyme disease and babesiosis disease manifestations. The mice were randomly divided into four experimental groups: (i) five uninfected, (ii) nine infected with N40 alone (one died during acclimatization period before infection), (iii) ten with N40 and *B. microti* together, and (iv) ten with *B. microti* alone. Mice infected with *B. microti* were monitored for parasitemia almost daily. To determine parasitemia at different days of infection, parasitized and total RBCs in 25 microscopic fields were counted in the stained blood smear from each infected mouse using oil immersion, 100× objective until parasitemia became undetectable by microscopic examination (21st day p.i.). Percent parasitemia was determined for each *B. microti*-infected and co-infected mouse throughout infection until euthanasia and is presented in Figure 1. Blood hemoglobin levels were also determined using a commercial kit (Hemocue^®^ Hb 201+ analyzer) according to the manufacturer’s instructions. During the acute phase of infection (11th day p.i.), mice were euthanized when *B. microti* parasitemia was between 15 and 20% while the experiment was concluded at 21st day of infection to evaluate the impact on both diseases after parasitemia became undetectable by microscopy. Before euthanasia, heparinized blood was collected to recover plasma.

### Determination of Tissue Colonization Levels and Disease Pathology

**FIGURE 2:**
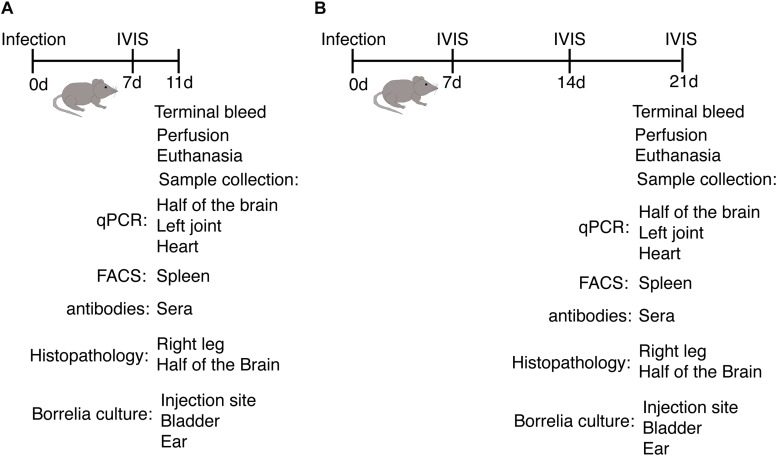
Outline of animal experiments to determine host response and disease manifestations after infection with *B. burgdorferi* N40 strain and/or *B. microti (Bm)*. **(A)** Experimental plan and samples analyses at the acute phase (11th day p.i.) of infection of C3H/HeJ female mice. **(B)** Evaluation of the impact of infection on the host and pathogens when the parasitemia becomes undetectable microscopically (21st day p.i.).

In each experiment, live spirochetes were recovered by culture using the skin at the injection site, ear, blood and urinary bladder in *Borrelia* medium while right joint and heart were processed for histological examination. For the acute phase of infection (11th day p.i.), blood was collected from the heart by cardiac puncture and two mice from each set were perfused after deep anesthesia to examine spirochetal colonization of brain (streamlined in Figure 2A). DNA was isolated from the left joint of mice in each experiment, from the ear only in the experiment concluded at 3 weeks of infection (not shown), and brain and heart in all other experiments. Burden of spirochetes was determined by employing our previously described duplex qPCR assay (Chan et al., 2013) and using CFX-96 Real-time system (Bio-Rad). Aseptically removed spleens were weighed, and splenocytes collected for flow cytometry as described previously (Djokic et al., 2018a, b). Sections of joints, spleen and hearts were mounted on slides and stained with hematoxylin and eosin (H&E) and used for histopathological examination. Sections of the heart and joints at 3 weeks of infection were evaluated by a pathologist board-certified by the American College of Veterinary Pathologists (KK) blinded to infection status according to established criteria. Two graduates of veterinary medicine (LA and VD) evaluated sections of spleen, and scored heart and joint samples at the acute phase of infection independently in a blinded manner and consensus results are presented here.

For immunostaining of *B. burgdorferi* in brains of infected mice, we used 1:100 dilution of FITC-Labeled BacTrace^®^ Anti-*B. burgdorferi* Antibody (Seracare) and 1:150 dilution of PE conjugated anti-mouse CD31 antibodies (Biolegend) for endothelial cell staining and employed our previously optimized procedures for *in vitro* assays or for spleen sections (Chan et al., 2016; Djokic et al., 2018a). Images were either acquired with a motorized Nikon Ti2 microscope using a 60× Plan APO, NA- 1.4 objective lens or were captured using the Nikon Eclipse Ti A1 scanning confocal microscope controlled by NIS-Elements software for image acquisition, processing, and analysis. The sections examined using Nikon Ti2 microscope were illuminated using a Lumencor Spectra X light engine and images were captured with a Hamamatsu ORCA Flash4.0 V3 sCMOS camera and Nikon NIS Elements software.

### Analyses of Splenic Cells by Flow Cytometry

Aseptically harvested spleens from infected mice were weighed, single cell suspensions of the splenocytes prepared, washed with PBS, and used for fluorescence-activated cell sorting (FACS) analyses using antibodies from Biolegend as previously described (Djokic et al., 2018a, b) with some modifications. Briefly, after counting live cells, splenocytes from each mouse were labeled with 1:50 dilution of anti-mouse CD45 coupled with PE (Biolegend) antibodies diluted in FACS buffer (PBS **+**5%FBS). Cell suspensions were incubated on ice in the dark for 30 min for staining. After washing three times with the buffer by centrifugation at 350 × *g* for 5 min each, cell pellets were suspended in 1 ml buffer and 5 samples from each mouse group pooled. Cell sorting was done using BD FACS AREA II (BD Biosciences) by first gating for appropriate cell size, then for DAPI negative live cells, followed by gating for PE positive cells. Then, CD45+ cells were stained for B cells with Brilliant violet 421 conjugated anti-mouse CD19 antibodies, T cells with PE/Cy7 conjugated anti-mouse CD3 antibodies, T helper cells with FITC conjugated anti-mouse CD4 antibodies and cytotoxic T cells with Alexafluor-700 conjugated anti-mouse CD8a antibodies, and macrophages with APC.Cy7 conjugated anti-mouse F4/80 antibodies (Biolegend) followed by FACS. Flow cytometry was conducted using BD LSRFortessa^TM^ X-20 (BD Biosciences) driven by software FACS DiVa (BD Biosciences). Acquired data was analyzed using FlowJo, Version 10.3 software.

### Humoral Response Determination

N40 culture was centrifuged at 4,000 × *g* for 15 min when density reached to 1–2 × 10^8^ spirochetes/ml and washed with PBS three times. The bacterial pellet was suspended in 0.1% B-per detergent (Thermo Fisher Scientific) containing PBS followed by sonication to lyse. After complete lysis was observed microscopically, total cell extract was passed through a 0.22 microfilter and the antigen preparation was stored at –20^∘^C until used for ELISA. ELISA plates were coated with 50 μl of *B. burgdorferi* N40 lysate (concentration adjusted to 0.3 mg/ml) and incubated at 37^∘^C overnight. Wells without protein coating (buffer only) were included as “No antigen” controls. Plasma samples recovered from uninfected and *B. burgdorferi* infected mice diluted at 1:5,000 were incubated with spirochetal antigen-coated wells for 1 h at room temperature. Eight replicates for each sample were used to ensure reproducibility. After incubation and washing three times for 5 min each with 0.5% Tween-20 containing PBS (PBST), plates were incubated with 50 μl of anti-mouse HRP conjugate for 1 h. The plates were then washed and bound antibodies detected using TMB substrate (KPL SureBlue). Absorbance was measured at OD_620_ using a SpectraMax M2 plate reader (Molecular Devices).

### Opsonophagocytosis of *B. burgdorferi*

To determine the changes in functional humoral immunity against *B. burgdorferi* N40 strain on co-infection with *B. microti*, 10^8^ spirochetes were suspended in 500 μl of binding medium containing 1:2 ratios of BSK-H (Sigma) and GHS (10 mM glucose+50 mM HEPES pH 7.0+10 mM NaCl). After preincubation of spirochetes with respective plasma samples diluted at 1:100 in J774A.1 macrophage medium (DMEM medium supplemented with 10% FBS), opsonophagocytosis was conducted as previously described (Chan et al., 2016). Thus, images of green extracellular and red internalized spirochetes after 2 h incubation to allow phagocytosis together with blue macrophages, labeled with wheat agglutinin lectin conjugated with Alexa fluor 647, were captured using the Nikon Eclipse Ti A1 scanning confocal microscope controlled by NIS-Elements software. Video of phagocytosed *B. burgdorferi* by N40 infected mouse plasma (Supplementary Video S1) was obtained using Leica TCS SP8 scanning confocal microscope with the system controlled by LAS X software for image acquisition, processing, and analysis.

### Statistical Analysis

All collected data were analyzed by Prism version 8.0 for Mac, GraphPad Software and comparisons made between groups using ANOVA and a two tailed unpaired student *t-*tests for unequal variance. Differences between paired groups with *p* < 0.05 were considered significant for a paired group comparison at 95% confidence interval.

## Results

### Effect of *B. burgdorferi* Strain N40 Co-infection on *B. microti*

In our experiments, mice infected with *B. microti* alone, and those co-infected with *B. microti* and N40, exhibited similar temporal patterns of parasitemia. In both cohorts, peak parasitemia was reached at 13th day post-infection (p.i.). Peak parasitemia levels were significantly higher in mice infected with *B. microti* (42.5 ± 5%) compared to co-infected mice (33.5 ± 1%) (Figure 1A). Increased parasitemia appears to have facilitated lysis of infected red blood cells (RBCs) and diminished hemoglobin levels in both sets of *B. microti* infected mice. Despite the lower peak parasitemia in co-infected mice, there was no statistically significant difference in hemoglobin levels compared to *B. microti* infected mice (Figure 1B). In both sets of mice, severe anemia was temporary and normal hemoglobin levels were restored within a few days of post-peak parasitemia. Higher parasitemia in *B. microti* infected mice was coupled with decreased erythrocyte density in blood compared to co-infected mice (Figure 1C, left versus right panel). Normal numbers of RBCs were restored in both sets of infected mice within 2 days of peak parasitemia (data not shown).

### Effect of *B. burgdorferi* and *B. microti* on Spleen and Splenocytes at Acute Phase of Infection

**FIGURE 3:**
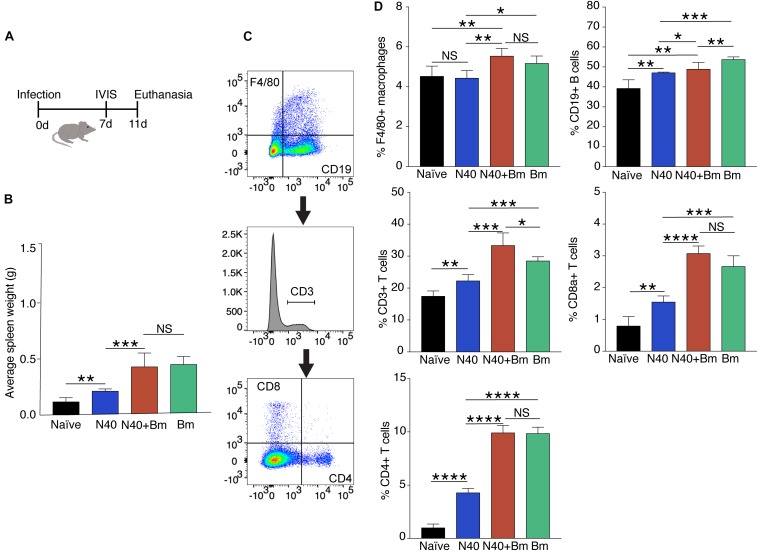
*Babesia microti (Bm)* infection caused splenic enlargement in C3H mice at the acute phase of infection and differentially increase splenocytes compared to N40-infected mice. **(A)** Overall experimental plan and samples analyses after infection of C3H/HeJ female mice. **(B)** Splenic weights were determined on day 11 p.i. Spleens of *Bm* infected and co-infected mice were significantly heavier than spleens from N40 infected mice, which showed moderate increase in weight compared to naïve mice. **(C)** Gating scheme used for FACS analysis of splenic CD45+ cells is shown. **(D)** Quantitative analyses of splenic B cells, T cells, CD4+ and CD8a+ cells, and macrophages in each group of mice. Each cell type is represented as percentage of total CD45+ splenic leukocytes (mean ± s.d., *n* = 5). Two-tailed unpaired student *t-*test for unequal variance between the paired groups was used to determine significance (NS, not significant, ^*^p < 0.05, ^∗∗^*p* < 0.01, ^∗∗∗^*p* < 0.001, ^****^p < 0.0001).

Experimental plan to determine the effect of infections at acute phase of infection is outlined in Figure 2A. As a major organ in the reticuloendothelial system, the spleen is involved in clearance of old, damaged or parasitized erythrocytes facilitating both blood filtration and resolution of parasitic diseases, including babesiosis (White et al., 1998). The spleen is a hematopoietic organ involved in homing of the lymphocytes and is also a reservoir of RBCs and monocytes. To determine the effect of co-infection on different organs, immune response and pathogenesis during the acute phase, infected mice were sacrificed on day 11 p.i. (Figure 3A). At this stage, infection with N40 alone caused a relatively small increase in the spleen weight while infection with *B. microti* either alone or together with N40 caused significant splenomegaly (Figure 3B). Analysis of total splenic CD45+ leukocytes during early infection by flow cytometry (shown for a representative mouse in Figure 3C) indicated the most significant increase in the F4/80 positive macrophages in *B. microti*-infected (*p* < 0.05, *df* = 8, *F* = 1.91) and co-infected (*p* < 0.01, *df* = 8, *F* = 1.72) mice compared to naïve mice, which suggests the role of macrophages is important in clearance of *B. microti* at the acute phase of infection. In contrast, macrophage number was not affected significantly in N40-infected mice (Figure 3D).

There was significant increase in percentage of the CD19+ B cells in *B. burgdorferi* infected mice relative to naïve mice at this early stage of infection demonstrating that induced expansion of these cells had started in these mice. However, increase in the percentage of CD19+ cells in *B. microti* infected (∼56%) and co-infected mice (∼51%) were even higher than in N40-infected (∼49%) and naïve (∼36%) mice (Figures 3C,D). *B. microti* infection also resulted in a significant increase in the T cell population in *B. microti* infected and co-infected mice, as reflected by the increase in the percent of CD3+ cells, the CD8a+ cells and CD4+ T cells (Figure 3D). This increase is consistent with previous observations in other parasitic diseases (Sponaas et al., 2006; Abel et al., 2012; Li et al., 2015). Significant increase in the T cells during *B. microti* infection from ∼17% in naïve to ∼29% in *B. microti* infection (*p* < 0.001, *df* = 6, *F* = 6.4) and ∼33% during co-infection (*p* < 0.01, *df* = 6, *F* = 9.99) compared to ∼22% in N40 infected mice emphasizes the role of these cells specifically during early stage of *B. microti* infection.

### Lyme Disease at Acute Phase of Infection

**FIGURE 4:**
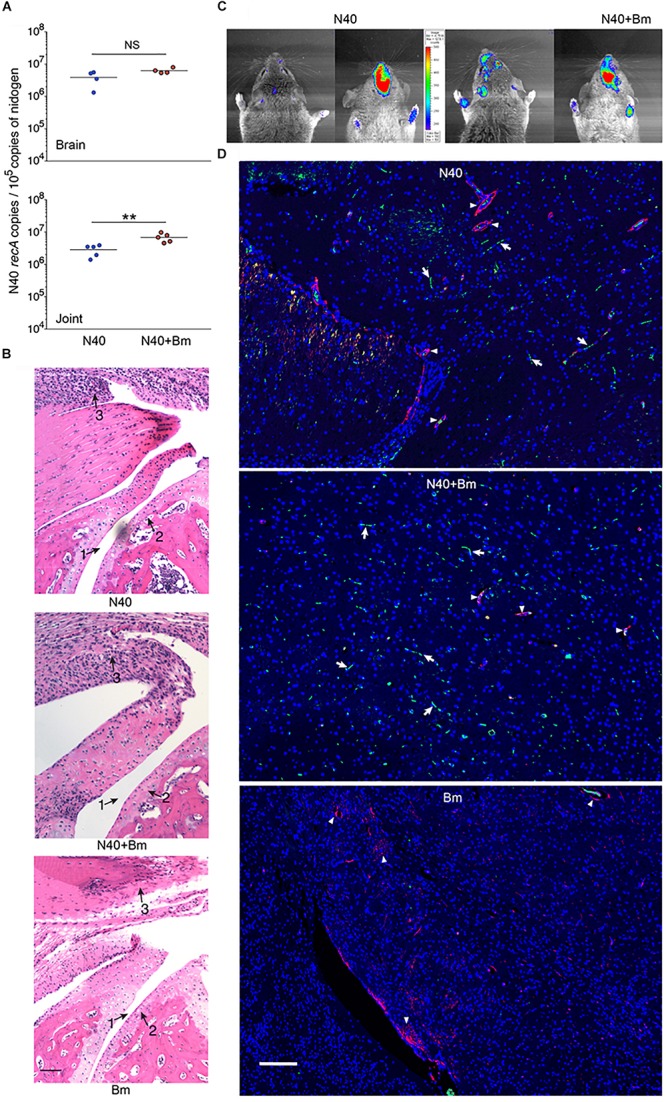
Effect of *B. microti* (*Bm*) and N40 co-infection on brain and joint colonization by *B. burgdorferi*, and their effect on joint inflammation during the acute phase of infection. **(A)** High numbers of spirochetes were observed in joints and brains of *B. burgdorferi* infected mice, while the N40 burden was significantly higher in joints of co-infected mice as determined by a two-tailed unpaired student *t-*tests for unequal variance between the paired groups (^∗∗^*p* < 0.01, *df* = 8, *F* = 3.60). **(B)** *B. burgdorferi* infection caused only mild joints inflammation during the acute phase of disease as indicated by change in synovial space (arrow 1), synovial hyperplasia and erosion of cartilage (arrow 2), and lymphocytic infiltration (arrow 3) while respective markers show higher inflammation in co-infected mice and no inflammation in *Bm*-infected mice. Bar represents 100 μm. **(C)** Images of the head region (left dorsal, right ventral) of N40-infected and co-infected mice captured by live imaging using IVIS-200 after i.p. injection of D-luciferin substrate showed an increase in bioluminescence, particularly in the frontal region of brain of co-infected mice. **(D)** Mice were deeply anesthetized, perfused with PBS and fixative before euthanasia. Brain sections were labeled with anti-*B. burgdorferi* antibodies conjugated to FITC and nuclei of host cells stained with DAPI. Endothelial cells were labeled using anti-CD31 antibodies tagged with PE (red) and are marked in the figure by arrowheads. Green spirochetes were detected in brain sections from N40 infected and co-infected mice (arrows) when the sections were examined using Nikon Ti2 microscope. *B. microti* infected mice used as a negative control did not show any spirochetes. Bar represents 100 μm.

At day 11 p.i., live imaging was used to detect dissemination of N40 from the site of infection. Bioluminescence was observed in the joints and head regions of all N40 infected and co-infected mice (not shown). Live spirochetes were recovered from the injection site, ear and bladder of all N40-infected and co-infected mice confirming a disseminated infection by *B. burgdorferi* (Table 1). To accurately quantify spirochetes burden in different tissues, N40 *recA* copy numbers were normalized to 10^5^ mouse nidogen copies in the duplex qPCR assay (Figure 4A). There was a high spirochete burden (>10^6^ *recA* copies/10^5^ nidogen copy number) in joints and brain of N40 infected or co-infected mice. High *B. burgdorferi* burden (>10^6^ spirochete *recA* copies/10^5^ mouse nidogen copies) is likely because the adaptive immune response was still not fully developed in these mice. N40 infected mice displayed inflammation in the tibiotarsus and their joints had significant infiltration of leukocytes compared to *B. microti* infected control mice (Figure 4B). Co-infection led to more pronounced inflammation with 2/5 co-infected mice displaying maximum (+++) arthritic severity and 3/5 displaying moderate (++) inflammatory arthritis. In the N40 infected group 4/5 mice showed moderate (++) and 1/5 mice displayed minimal (+) inflammation (Figure 4B and Table 2). None of the N40-infected or co-infected mice displayed any apparent carditis (data not shown).

**TABLE 1:** Cultivation of *B. burgdorferi* from different organs of the infected mice in BSKII medium containing 6% rabbit serum.

	**N40**			**N40+Bm**		
**Infection stage**	**Ear**	**Injection Site**	**Bladder**	**Ear**	**Injection Site**	**Bladder**
11 days	5/5	5/5	5/5	5/5	5/5	5/5
21 days	9/9	9/9	9/9	10/10	10/10	10/10

**TABLE 2:** Histopathological scoring of joints of infected mice at two points of infection.

	**No of mice with each histological score**							
**Experimental groups**	**Knee**			Tibiotarsus^*^				
**Score**	**−**	**±**	**+**	**−**	**±**	**+**	**+⁣+**	**+⁣+⁣+**
Day 11th N40	1	2	2	0	0	1	4	0
N40+Bm	1	0	4	0	0	0	3	2
Bm	5	0	0	5	0	0	0	0
Day 21st N40	2	2	5	0	1	3	5	0
N40+Bm	2	1	7	0	0	2	4	4
Bm	9	1	0	9	1	0	0	0

^*^Scoring of severity of arthritic manifestation of knee was negative (−), equivocal (±) or positive (+). Tibiotarsus inflammation ranged from negative (−) to severe (+⁣+⁣+) in B. burgdorferi infected/co-infected mice based upon; (i) synovial hyperplasia (ii) erosion of cartilage, (iii) increase in lymphocytic infiltration and (iv) change in synovial space compared to the naïve mice or mice infected with B. microti alone.

Earlier studies that reported dissemination of N40 to the mouse brain did not conduct a thorough investigation of brain colonization. Anecdotally, many researchers do not now believe that *B. burgdorferi* can invade mouse brain despite this being reported by Barthold et al. (1992). Light emission by our bioluminescent N40 focused in the head region by IVIS (Figure 4C) indicated that live spirochetes were likely present in brain. After removing frontal brain region for immunohistology, we confirmed colonization in remaining part of the brain by N40 by qPCR (Figure 4A). *B. burgdorferi* uses blood as transient conduit for dissemination. To minimize the presence of spirochetes in the vasculature, deeply anesthetized mice were perfused with PBS and fixative before euthanasia. We removed a small frontal section of brain (6–8 mm in thickness from chiasma opticum) from perfused mice for immunostaining to detect *B. burgdorferi.* Fixed brain sections from frontal region of N40-infected and co-infected perfused mice were immunostained with *B. burgdorferi* specific antibodies conjugated to FITC at the acute phase of infection, i.e., 11th day p.i. (Figure 4D). In both; N40 infected and co-infected groups of mice, green spirochetes were detected in brain tissue in the frontal region, similar to that shown previously in parenchyma of *B. burgdorferi* infected rhesus macaque brain sections (Ramesh et al., 2008, 2009). The presence of N40 was not restricted to vasculature, as demonstrated by spirochetes location in brain that is distant from red-labeled CD31, a marker for endothelial cells (Figure 4D). *B. microti* infected mice used as negative controls showed no green spirochetes in brain section, as expected (Figure 4D). Thus, in addition to live imaging and qPCR results, we examined several forebrain sections (frontal lobe) from perfused animals to confirm *B. burgdorferi* presence in the brains beyond vasculature and to show brain parenchyma colonization.

### Impact of *B. microti* Infection on Splenomegaly and Splenocytes Post-parasitemia

**FIGURE 5:**
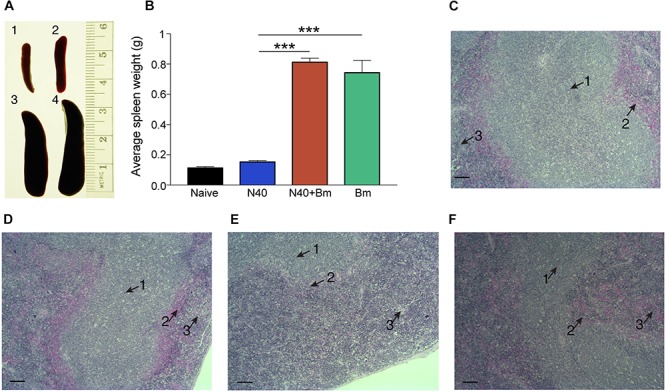
*Babesia microti* (*Bm*) infection caused pronounced splenomegaly affecting splenic architecture in C3H mice at day 21 p.i. **(A,B)** A significant but moderate increase in spleen size is observed in N40 infected mice (2) compared to naïve mice (1) while very pronounced splenomegaly is observed in *Bm* infected (3) mice (^∗∗∗^*p* < 0.001, *df* = 3, *F* = NA) and co-infected (4) mice (^∗∗∗^*p* < 0.001, *df* = 5, *F* = NA). **(C,D)** H&E stained spleen sections displayed normal architecture with a clear demarcation between the white and red pulp (arrows 1 and 3, respectively) and marginal zone (arrow 2), in uninfected **(C)**, and N40-infected **(D)** mice. **(E,F)** Demarcation between red and white pulp was indistinguishable in spleens of co-infected **(E)**, and *B. microti* infected **(F)** mice. Bar in microscopic images represents 100 μm.

Outline of the experiment and samples analyses post-parasitemia (day 21 p.i.) is provided in Figure 2B. Splenocytes proliferation has been reported to occur in response to *B. burgdorferi* infection but spleens of N40 infected mice were only slightly larger than naive mice while *B. microti* infected mice consistently demonstrated pronounced splenomegaly at 21st day p.i. (Figures 5A,B). More than 3-fold increase in spleen weight was observed in *B. microti* infected (*p* < 0.001, *df* = 5, *F* = 1.93) and co-infected mice (*p* < 0.0001, *df* = 7, *F* = 3.01) compared to N40 infected mice. The enlargement of spleens from *B. microti-*infected mice could be attributed to the increased hematopoiesis support it provides, while its dark coloration could result from *B. microti*-mediated RBCs lysis and erythrophagocytosis by macrophages. Histopathological examination of spleen sections demonstrated a clear demarcation between the red and white pulp regions, the marginal zone and trabeculae in N40-infected mice that was similar to naive mice (Figures 5C,D). In spleens from *B. microti*-infected and co-infected mice, the demarcation zone between red and white pulp was indistinguishable (Figures 5E,F). Co-infected animals demonstrated cellular proliferation, general infiltration of white cells and expansion of the red pulp (Figure 5E) while *B. microti*-infected mice displayed overall enlargement of the white pulp (Figure 5F).

### Effect of *B. microti* on Splenic Immunity After Parasitemia Resolution

**FIGURE 6:**
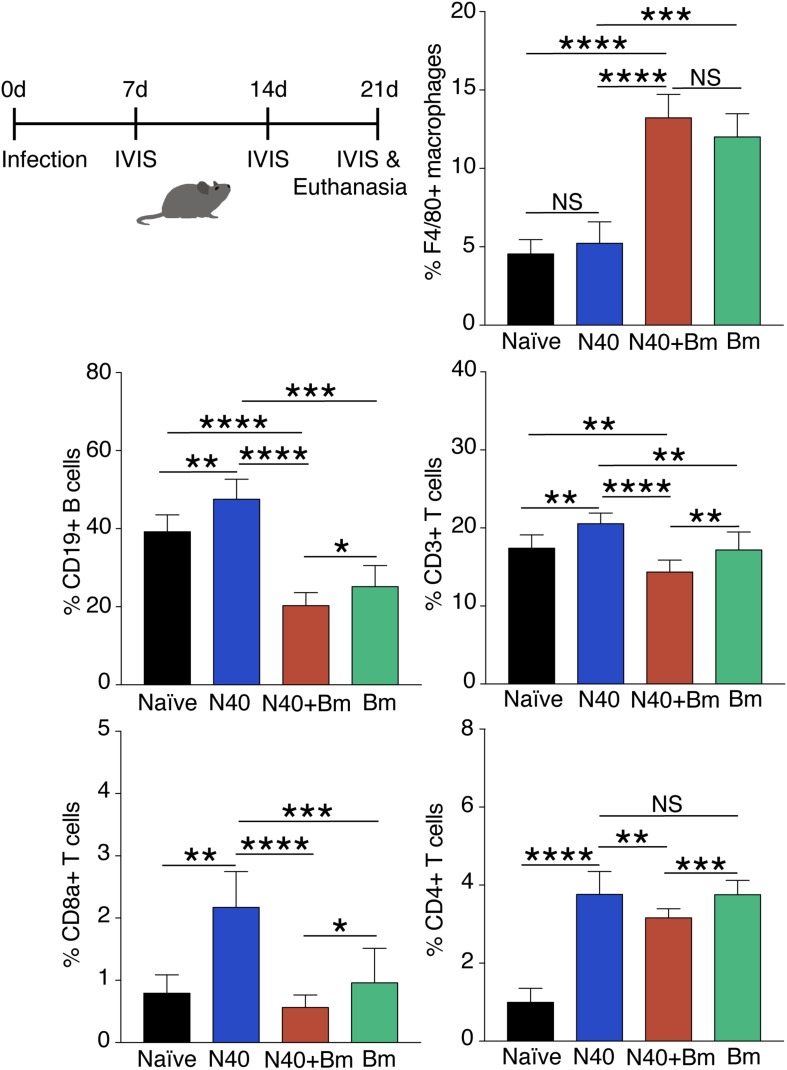
Flow cytometry analysis of splenic leukocytes from infected mice at day 21 p.i. Percentage of each cell type in each mouse is calculated using total CD45+ cells and data presented as mean ± s.d. Increase in F4/80+ macrophage percentage remained significantly higher only in co-infected mice (*n* = 10) compared to the naïve, uninfected mice (*n* = 5). Significant but only moderate increase in T and B cells was observed in *Bm* infected mice (*n* = 10). Increase in CD19+B cells, total CD3+ T cells, and CD8a+ cells in N40 infected (*n* = 9) and *Bm* infected mice was observed at this stage while significant reduction in co-infected mice occurred compared to respective cells in mice infected with each pathogen individually. Increases in CD4+ T cells relative to naïve mice in *Bm*-infected and N40 infected mice were higher than in co-infected mice. Each bar represents the mean ± s.d. (NS, not significant, ^*^p < 0.05, ^∗∗^*p* < 0.01, ^∗∗∗^*p* < 0.001, ^****^p < 0.0001).

To determine longer-term effects of *B. microti* infection on the spleen, using the gating scheme presented in Figure 3C, we examined changes in splenic leukocyte sub-populations after resolution of parasitemia at day 21 p.i. The percentage of macrophages remained significantly higher in co-infected mice (∼13%, *p* < 0.0001, *df* = 12, *F* = 2.69) and in *B. microti* infected mice (∼12%, *p* < 0.0001, *df* = 12, *F* = 2.68) relative to naïve mice (∼4.5%) even at this stage of infection while their percentage in N40 infected mice (∼5%) was not significantly different from controls (Figure 6). The percentage of CD19+ B cells was significantly higher at this stage of infection primarily in mice infected with *B. burgdorferi* (∼48%, *p* < 0.01, *df* = 12, *F* = 1.38) compared to naïve mice. In *B. microti* infected mice, percentage of B cells was reduced significantly (∼25%, *p* < 0.001, *df* = 13, *F* = 1.54) compared to naïve mice (39%). A marked reduction in B cells in co-infected mice was also observed relative to naïve (∼20%, <0.0001, *df* = 13, *F* = 1.69) and N40-infected (*p* < 0.0001, *df* = 17, *F* = 2.33) mice (Figure 6 and Table 3) at day 21 p.i. Thus, percentage of B cells in spleen appeared to be consistently lower in co-infected mice compared to *B. microti* infected mice.

**TABLE 3:** Analyses of splenic macrophage, B, and T cells by flow cytometry at 11th day and 21st day post-infection.

**11th day p.i.**					**21st day p.i.**			
	**Splenocytes**		^*^Total No. × 10^2^	**Std. Dev.**	**Splenocytes**		^*^Total No. × 10^2^	**Std. Dev.**
N40	CD45		500	0	CD45		500	0

		F4/80 macrophages	22.2	1.9		F4/80 macrophages	26.1	6.8
		CD19 B cells	642.0	40.8		CD19 B cells	237.9	25.4
		CD3 T cells	108.8	9.3		CD3 T cells	102.7	6.9
		CD8a	7.6	1.1		CD8a	10.9	2.9
		CD4	21.4	2.1		CD4	18.8	2.9
N40+Bm	CD45		500	0	CD45		500	0
		F4/80 macrophages	27.7	1.9		F4/80 macrophages	66.2	7.5
		CD19 B cells	254.4	8.6		CD19 B cells	101.5	16.6
		CD3 T cells	166.5	19.8		CD3 T cells	71.8	7.7
		CD8a	15.4	1.2		CD8a	2.8	1.0
		CD4	49.6	3.5		CD4	15.8	1.2
Bm	CD45		500	0	CD45		500	0
		F4/80 macrophages	25.9	1.9		F4/80 macrophages	60.0	7.5
		CD19 B cells	279.1	10.7		CD19 B cells	125.9	26.8
		CD3 T cells	142.5	6.7		CD3 T cells	85.9	11.6
		CD8a	113.9	1.4		CD8a	4.8	2.8
		CD4	49.2	3.0		CD4	18.8	1.8
Naïve	CD45		500	0	CD45		500	0
		F4/80 macrophages	22.7	2.4		F4/80 macrophages	22.7	4.6
		CD19 B cells	181.5	27.1		CD19 B cells	196.1	21.6
		CD3 T cells	92.2	6.6		CD3 T cells	87.1	8.4
		CD8a	3.9	1.5		CD8a	3.2	2.2
		CD4	4.9	1.8		CD4	4.9	1.8

^*^Average of estimated cell numbers for five in naïve mice and in each infection group.

We found that percentage of total T (CD3+) cells in *B. microti* infected mouse spleens remained comparable to naïve mice (∼17% each), increase in percentage of total T cells, CD8+ cells, and CD4+ cells was noted to be high in response to infection with *B. burgdorferi* alone similar to that observed for B cells (Figure 5). Thus, percentage of the CD3+ T cell was significantly lower in co-infected mice (*p* < 0.0001, *df* = 17, *F* = 1.23) with an average of ∼14% T cells compared to ∼21% in N40 infected mice despite pronounced splenomegaly observed in the *B. microti* infected and co-infected mice (Figure 6). Overall, percentage of total splenic B and T cells were significantly lower in *B. microti* infected relative to N40 infected mice in multiple experiments indicating that *B. microti* infection (with or without *B. burgdorferi* infection) stimulated proliferation of splenic B and T cells at lower levels than that by N40 infection at a time point when adaptive immune response is usually established (21st day p.i.). In fact, *B. microti* appeared to suppress adaptive immune response (Figure 6) since percentage of the splenic CD8a+ cells also diminished in co-infected mice (∼0.6%) compared to N40 infected mice (∼2%) and only increased slightly in *B. microti* infected (∼1%) relative to naïve mice (∼0.8%). There was a significant increase in percentage of CD4+ cells in all three groups of infected mice compared with naïve mice (∼1%) such that their levels were comparable in N40 infected and *B. microti* infected mice (∼3.8% each) while percentage of these cells was significantly lower (∼3.2%) in co-infected mice compared to N40 infected (*p* < 0.01, *df* = 17, *F* = 6.23) and *B. microti* infected (*p* < 0.001, *df* = 18, *F* = 2.41) mice (Figure 6).

### Immunomodulation of Humoral Response by *B. microti*

**FIGURE 7:**
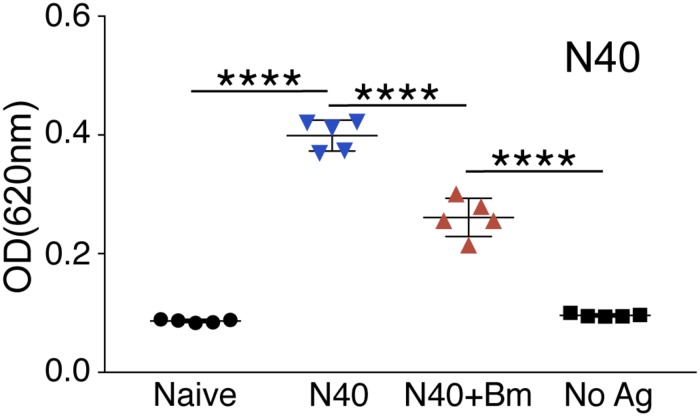
Determination of the specific antibody response in N40 infected and co-infected mice at day 21 p.i. ELISA analysis using N40 protein extract probed with pooled plasma of either N40 infected or co-infected mice indicated a significant reduction (^****^p < 0.0001, *df* = 8, *F* = 1.55) in the specific antibodies against *B. burgdorferi* in co-infected mice. No reactivity was observed in the negative control (No Ag) or using naïve mice plasma.

The significant decrease in the percentage of B and T cells (*p* < 0.0001) in spleen of co-infected mice compared to N40-infected mice as well as naïve mice (Figure 6) suggests that *B. microti* infection either leads to depletion of T and B cells or inhibits their proliferation. We further determined if *B. microti*-mediated depletion in B and T helper cells affected the antibody response against *B. burgdorferi*. We used ELISA to quantify antibody responses, in co-infected and N40-infected mice, using total *B. burgdorferi* protein extract as antigen. The antibody response in co-infected mice was significantly attenuated compared to N40-infected mice (Figure 7). The decreased antibody response in co-infected mice is consistent with diminished levels of the B cells in these mice.

### *B. microti* Enhances *B. burgdorferi* Survival and Increases Lyme Disease Severity

**FIGURE 8:**
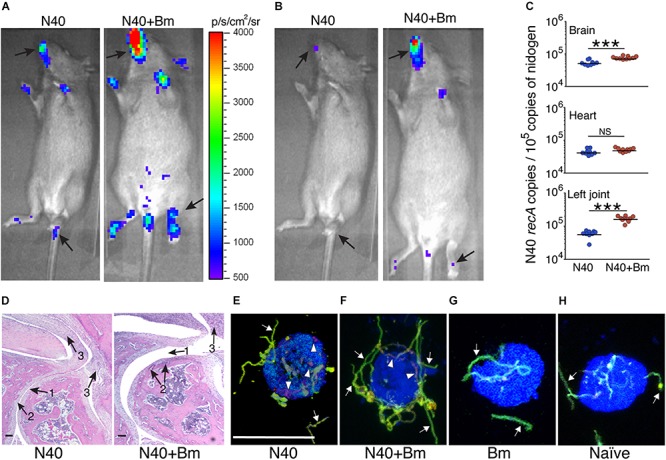
Increased colonization of organs by N40 and exacerbation of Lyme disease in mice by co-infection with *B. microti* (*Bm*). **(A)** Representative real-time images using IVIS-200 displaying higher spirochetal burden in co-infected than N40-infected mice at 2 weeks, and **(B)** 3 weeks of infection. **(C)** Burden of N40 in tissues determined using duplex qPCR assay with horizontal lines representing the mean N40 *recA* copy number. Joint and brain of co-infected mice showed significantly (^∗∗∗^*p* < 0.001) higher spirochete burden as compared to mice infected with N40 alone while *B. burgdorferi* burden in hearts was not significantly different (NS). **(D)** More severe arthritis manifested by change in synovial space (arrow 1), synovial hyperplasia and erosion of cartilage (arrow 2), and higher lymphocytic infiltration (arrow 3) were observed in co-infected mice as compared to the N40-infected mice. **(E)** Opsonophagocytosis of N40 by mouse J774.1 macrophages was observed after 2 h of co-incubation of plasma from N40-infected mice with spirochetes such that macrophages showed significant phagocytosis detected as red, internalized bacteria (arrowheads) and some green extracellular spirochetes (arrows). The macrophages are marked blue. **(F)** Although phagocytosis occurred after opsonization of N40 with plasma from co-infected mice, it showed significantly lower internalized spirochetes compared to those using plasma from N40-infected mice **(E)**. **(G)** Incubation of *B. burgdorferi* using plasma from *B. microti* infected mice, and **(H)** uninfected, naïve mice showed no phagocytosis of the spirochetes after 2 h of co-incubation of *B. burgdorferi* with macrophages. Bar represents 100 μm in Figures 7E–H.

Increased bioluminescence in co-infected mice suggested a higher bacterial burden in this group compared to N40-infected mice at 2 weeks of infection (Figure 8A). The increased bacterial burden in co-infected mice was also observed at day 21 p.i. (Figure 8B). Live spirochetes could be recovered from all tissues of N40 infected and co-infected mice (Table 1). For further examination, brain, heart and joint tissues were collected from mice at day 21 p.i. Spirochete burden in different tissues was determined by qPCR and histopathological evaluations of joint and heart sections were also conducted. Significantly higher levels of N40 burden in the brains and joints of co-infected mice compared to N40-infected mice (Figure 8C) confirmed the live-imaging results. Although the colonization level in hearts was slightly higher in co-infected mice, the difference was not statistically significant (Figure 8C). Inflammatory arthritic manifestations in the tibiotarsus were scored to be significantly higher (*p* = 0.045) in co-infected mice at day 21 p.i., as depicted by synovial membrane hyperplasia, erosion of articular cartilage, lymphocytic infiltration in synovial membranes, and widening of synovial space in co-infected mice (Figure 8D). Indeed, 8/10 co-infected mice showed moderate to severe (++ to +++) arthritis while none of the N40 infected mice showed severe arthritis and only 5/9 showed moderate (++) inflammatory arthritis (Table 2). Hence, increased colonization of joints by N40 in co-infected mice resulted in more pronounced inflammatory Lyme arthritis. Cardiac inflammation determined by histological scoring was comparable in N40 infected and co-infected mice indicating correlation of inflammatory disease with spirochete burden in heart (data not shown). Lower *B. burgdorferi* numbers at day 21 p.i. compared to day 11 p.i. (Figure 4A versus Figure 8C) likely represented partial clearance of spirochetes by an adaptive immune response. Overall, severity of inflammatory Lyme disease correlated with the N40 load in the respective tissues and both spirochetes burden and inflammatory responses were amplified by co-infection with *B. microti*.

To determine the role of N40-specific antibodies in functional immunity, we further conducted phagocytosis following opsonization with pooled plasma from N40 infected and co-infected mice (Figures 8E,F). Pooled plasma from the naïve mice and *B. microti* infected mice served as the negative controls (Figures 8G,H). When opsonized with pooled plasma from N40 infected mice, all macrophages (100%) showed red phagocytosed *B. burgdorferi* (Figure 8E and Supplementary Video S1). Opsonization with plasma from co-infected mice led to phagocytosis by 60% of macrophages. Only extracellular spirochetes were observed attached to the remaining (40%) macrophages (Figure 8F and data not shown) indicating diminished inducible functional immunity in these mice. No opsonophagocytosis was observed in the negative controls (Figures 8G,H) indicating that this assay determined the specific functional immune response in infected/co-infected mice.

**FIGURE 9:**
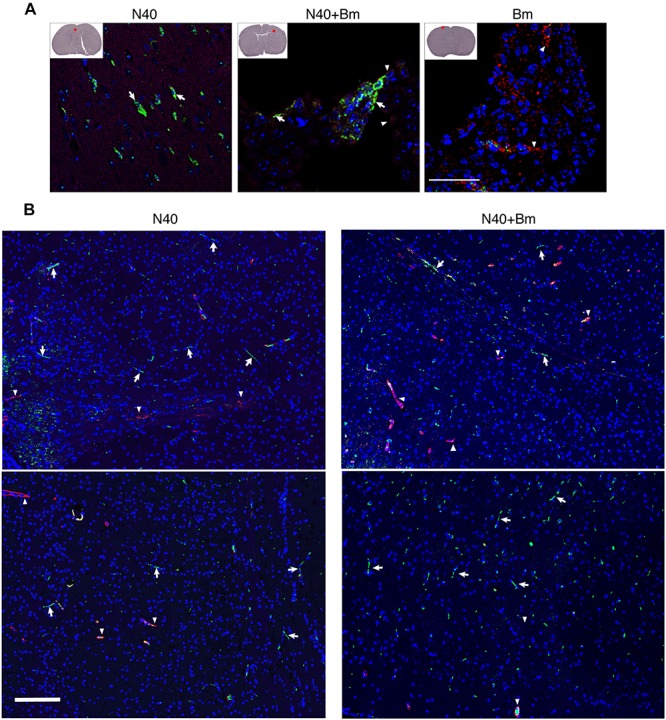
*Borrelia burgdorferi* N40 strain infection with or without *B. microti* co-infection resulted in colonization of the forebrain at day 21 p.i., as shown in multiple sections by immunostaining. **(A)** Deeply anesthetized mice were perfused with PBS and fixative before euthanasia. Brain sections were labeled with anti-*B. burgdorferi* antibodies conjugated to FITC (white arrows) and endothelial cells using anti-CD31 antibodies tagged with PE (red), marked by arrowheads. The nuclei of the host cells are stained blue by DAPI. Aggregates of green spirochetes (arrows) were detected in brain sections from N40 infected and co-infected mice when examined by Nikon Eclipse Ti A1 scanning confocal microscope. Control *B. microti* infected mice brain did not show any spirochetes. **(B)** Additional sections of brains of mice infection with N40 alone or co-infected with *B. microti* also showed presence of the spirochetes when the sections were examined using Nikon Ti2 microscope illuminated using a Lumencor Spectra X light engine and images captured with a Hamamatsu ORCA Flash4.0 V3 sCMOS camera and Nikon NIS Elements software. Arrows mark green spirochetes in the image while arrowheads depict red endothelial cells. Bar represents 100 μm.

Finally, similar to that observed during the acute phase of infection, we examined brain sections of mice perfused with PBS before euthanasia at day 21 p.i. after staining for host nuclei with DAPI, embedded spirochetes with anti-*B. burgdorferi* FITC conjugate, and endothelial cells with PE conjugated anti-mouse CD31 antibodies (Figure 9). N40 spirochetes were either detected as clumps (Figure 9A) or individually at 3 weeks p.i. (Figure 9B).

## Discussion

Our study demonstrates the interactions and effects of two tick-borne pathogens on each other and on the susceptible host. We examined *B. burgdorferi* and *B. microti* infection at the acute phase of infection (before peak parasitemia and development of adaptive immune response) and immediately following resolution of *B. microti* parasitemia. We observed a lower peak parasitemia during co-infection (Figure 1). Previously, higher *B. microti* parasitemia was observed after tick-transmission in outbred, *Peromyscus leucopus* mice when animals were co-infected with another strain of *B. burgdorferi*, B348, compared to the animals infected by *B. microti* alone (Dunn et al., 2014). B348 strain causes disseminated infection in humans (Hanincova et al., 2013) but is a slow disseminator in mice (Hanincova et al., 2008). Differences in our results and outcomes in studies by Dunn et al. (2014) could be either due to the mode of infection (tick versus needle), genotypes of pathogens, or genetic differences between *Peromyscus* versus *Mus musculus* C3H mouse strain used. Supporting this premise, stimulation of the innate immune response against invasive N40 strain could possibly resolve babesiosis while it is likely not sufficient against the less fit *B. burgdorferi* B348 strain in *P. leucopus*, thus resulting in higher Babesia parasitemia in co-infected than *B. microti* infected animals (Dunn et al., 2014). In any case, higher parasitemia in *P. leucopus* could facilitate acquisition of *B. microti* from this co-infected animal-reservoir host in nature by tick vector (Diuk-Wasser et al., 2016). High parasitemia with Apicomplexan protozoan that infect RBCs, such as malaria causing *Plasmodium* species, is followed by lysis of erythrocytes causing anemia (Shabani et al., 2017). Hematologic abnormalities, such as anemia and thrombocytopenia are also associated with babesiosis in humans, often requiring blood transfusion and even hospitalization (White et al., 1998; Hatcher et al., 2001; Joseph et al., 2011). *B. microti* infection in mice in this study (Figure 1C) and previously reported (Coleman et al., 2005), and infection of gerbils by *B. divergens* (Dkhil et al., 2010) resulted in overall reduction of erythrocytes in blood. Thus, our results here reflect the effect of *Babesia* infection in the susceptible hosts.

The spleen is suggested to be an important lymphoid organ that produces plasma cells, which are the major producer of antibodies during protozoan infection (Bermejo et al., 2011). Previous studies have shown that humoral immune response against parasites causing Chagas disease and malaria are delayed or abrogated due to splenic B cell apoptosis and depletion (Muxel et al., 2011; Obishakin et al., 2014). Furthermore, severe babesiosis in splenectomized patients results in high morbidity and even mortality indicating a critical role of the spleen in resolution of *Babesia* infection. Thus, splenomegaly and alteration in spleen architecture in *B. microti* infected mice as observed in our study are consistent with reports on other parasitic diseases (Kafetzis, 2003; Dkhil et al., 2010; Wilson et al., 2011; Kuna et al., 2015). Movement of activated marginal zone B cells and dendritic cells to the T cell zone help presentation of antigen directly and therefore, activation of T cells followed by their migration to the edge of the follicles (Vannier and Krause, 2012). These changes can possibly obliterate the demarcation between the red and white pulp, as we observed (Figure 5). In humans, babesiosis can be a life-threatening disease in asplenic individuals, further emphasizing the importance of the spleen in babesiosis resolution (Krause et al., 2008; Raffalli and Wormser, 2016). Even after elimination of the parasite, recovery of internal organs including spleen could lag behind, prolonging illness. Unlike humans, death has not been reported in mice due to *B. microti* infection. We also did not observe any visual differences in vitality of *B. microti* infected versus co-infected mice.

Innate immunity was reported to be critical for determining the fate of *Babesia* infection in mice (Aguilar-Delfin et al., 2001). In mice, the spleen is a major reservoir of undifferentiated monocytes that can be differentiated into macrophages and dendritic cells *in vitro*. It is conceivable that infection with *B. microti* stimulates these cells to develop into macrophages, which then facilitate clearance of the infected erythrocytes as we showed previously (Djokic et al., 2018a, b). Depletion of macrophages using drugs at different stages of *B. microti* infection resulted in significant increases in parasitemia and caused mortality in the mice (Terkawi et al., 2015). Despite development of high *B. microti* parasitemia levels, anti-inflammatory response could prevent death in our experiments unlike that reported for highly infectious *B. duncani* WA-1 strain in mice and hamsters (Dao and Eberhard, 1996; Hemmer et al., 2000). Mortality due to WA-1 strain is associated with the high levels of IFN-γ and TNF-α in spleen and lungs, heavy intravascular hemolysis, and pronounced vascular stasis with multi-organ failure (Dao and Eberhard, 1996; Hemmer et al., 2000). Our findings suggest that unlike *B. duncani* WA-1 strain, proinflammatory immunological response to *B. microti* is more subdued, protecting animals from death.

Our results agree with the previous report that young C3H mice show pronounced inflammatory Lyme arthritis manifestations (Barthold et al., 1990). Severity of Lyme arthritis and carditis in C3H mice correlates with the *B. burgdorferi* burden (Yang et al., 1994; Ma et al., 1998; Brown et al., 2001; Thomas et al., 2001; Parveen et al., 2006; Sahay et al., 2011; Schlachter et al., 2018). Several host factors contribute to inflammatory disease. Previous histopathological examination of *B. burgdorferi*-infected mice showed infiltration of innate immune cells, predominantly neutrophils, at sites of inflammation in the joints (Barthold et al., 1990, 1992; Ruderman et al., 1995; Sahay et al., 2011). In addition, depletion of CD8+ cells using antibodies helped resolution of ankle swelling in C3H/HeJ mice, indicating that these cells exacerbate inflammatory Lyme arthritis (Lasky et al., 2016). Other factors, such as the increase in proinflammatory cytokines production, also contribute to inflammatory Lyme disease. For example, reduction in *B. burgdorferi*-specific proinflammatory cytokine production in infected C3Hgld mice, due to the presence of a non-functional mutation in Fas ligand (FasL), caused diminished inflammatory response and less severe Lyme arthritis even though spirochete burden in C3Hgld mice was similar to C3H mice (Shi et al., 2006). Fas is reported to be expressed at high levels in macrophages, dendritic cells, fibroblasts, and lymphocytes present in inflamed synovium, while FasL is expressed in macrophages and γδ T cells of synovium (Perlman et al., 1999; Roessner et al., 2003; Ma et al., 2004; Shi et al., 2006). Although we did not use mice defective in a particular cell type or immuno-depleted our mice for any particular cell type, we observed a high level of infiltration of leukocytes in the inflamed joints of *B. burgdorferi* infected and co-infected mice (Figure 8D).

Unlike a previous report that *B. microti* and *B. burgdorferi* have independent courses of infection in co-infected mice (Coleman et al., 2005), we observed that *B. microti* infection has a significant impact on increasing *B. burgdorferi* survival and tissue colonization. B cells are important professional antigen presenting cells, display regulatory functions through cytokine production and are critical for humoral immunity due to their production of protective antibodies. Subversion of different B-cell subsets during parasitic and viral infections was reviewed recently (Borhis and Richard, 2015). Significant reduction in total B and T cells was also reported after infection with malaria parasite, *P. falciparum* in patients compared to uninfected controls (Kassa et al., 2006). Significantly lower numbers of splenic B and T cells after *B. microti* co-infection in our study agrees with these findings. The impact of destabilization of B cell numbers by *B. microti* is also reflected in the attenuated antibody response against *B. burgdorferi* during co-infections (Figure 7). Antibodies play an important role in clearance of extracellular *B. burgdorferi* by engaging different effector mechanisms, such as complement activation, neutralization, and opsonization, which results in phagocytosis facilitated by interaction of the Fc-region of antibodies and Fc-receptors on the professional phagocytes. In fact, adaptive immune responses involving both B and T cells have been implicated in resolution of inflammatory Lyme disease in mice (Barthold et al., 1992; McKisic and Barthold, 2000; McKisic et al., 2000; Bockenstedt et al., 2001). Both splenic B cell populations and serum immunoglobulin levels are elevated in response to *B. burgdorferi* infection. Immunological memory persists for a long period after antibody maturation. Therefore, *B. burgdorferi*-specific antibodies are important for clearance of the spirochetes in animals by opsonophagocytosis (Belperron et al., 2014). However, residual spirochetes remain in various organs of mice (Barthold et al., 1990). Overall, diminished functional humoral immunity due to *B. microti* infection specifically against *B. burgdorferi*, as determined by opsonophagocytosis, could prolong survival of Lyme spirochetes in the co-infected mice (Figures 4, 8). Alternatively, induction and preferential expression of the specific genes in N40 during co-infections could facilitate survival and persistence of spirochetes in tissues. Reported changes in essential gene expression in pathogens during co-infections support this hypothesis (Steere et al., 2011; Wu et al., 2015; Ibberson et al., 2017).

*Borrelia burgdorferi* stimulates splenic B and T development at 3 weeks of infection which is suppressed by *B. microti* and results in overall reduction in the humoral immunity, increases tissue colonization by *B. burgdorferi* and facilitates persistence of inflammatory Lyme arthritis in co-infected mice. Somewhat higher levels of infiltration of leukocytes in the co-infected mice could also contribute to increased joints inflammation (Barthold et al., 1990, 1992; Ruderman et al., 1995; Lasky et al., 2016). The effect of infection with N40 on *B. microti* was subtle, but we consistently observed diminished peak parasitemia in the co-infected mice. Our results here reflect outcome of simultaneous co-infections with two tick-borne pathogens and may differ when infection with *B. microti* and *B. burgdorferi* occurs in sequential manner. It is of great interest to us to examine the impact of *B. microti* prior presence in animals on a follow up infection by *B. burgdorferi* and vice versa on the host. This will be focus of our future studies. Despite some differences observed in severity of diseases in mice and humans during co-infection with *B. burgdorferi* and *B. microti*, our results indicate that a thorough investigation using susceptible mice can provide insights into their respective pathogenesis. In addition, a better understanding of pathogenesis also requires a careful examination of the mechanisms involved in the development and stimulation of splenic B and different T cell populations at different stages of infection using the susceptible C3H animal model system developed here. Furthermore, mechanisms involved in reduction of B and to some extent T cells need to be determined to fully understand the impact of co-infection with *B. burgdorferi* and *B. microti*. Our future studies will address these questions.

## Conclusion

Our studies indicated that during co-infection of susceptible C3H mice with tick-borne pathogens, potential stimulation of the innate immune response by *B. burgdorferi* attenuate *B. microti* parasitemia while changes in symptoms of babesiosis were not discernible. However, in our model, *B. microti* suppressed adaptive immune response triggered by *B. burgdorferi* infection such that diminished splenic B and T cells populations were reflected by overall reduction in the specific functional humoral immunity against both pathogens. As a consequence, *B. burgdorferi* persists at higher levels in tissues causing more severe Lyme disease in the susceptible C3H mice.

## Data Availability

All data generated or analyzed during this study are included in this published article (and its Supplementary Information File).

## Ethics Statement

The Institutional Animal Care and Use Committee (IACUC) members reviewed and approved the protocol number PROTO201702491 entitled, “Spirochetes and tick-borne pathogens,” of the corresponding author to conduct this study at Rutgers New Jersey Medical School following guidelines of the Animal Welfare Act, The Institute of Laboratory Animal Resources Guide for the Care and Use of Laboratory Animals, and the Public Health Service Policy that are fully adopted at the Rutgers University.

## Author Contributions

NP conceived and designed the experiments. PB provided training to LA in microscopy. VD, LA, SP, and SS performed the experiments. LA and VD conducted imaging of H&E stained organs sections, and performed analysis of heart and joints (at acute phase), and spleen and liver independently at three weeks post-infection. KK performed histopathological analysis and inflammation scoring of heart and joints in a blinded manner at three weeks of infection.

## Conflict of Interest Statement

The authors declare that the research was conducted in the absence of any commercial or financial relationships that could be construed as a potential conflict of interest.

## Supplementary Material

VIDEO S1 3D visualization of the phagocytosis of *B. burgdorferi* using pooled N40-infected mice plasma for opsonization. Rotating angle view in the space demonstrates surface labeling of J774.1 macrophages (blue), extracellular B. burgdorferi (green), and B. burgdorferi internalized by the macrophages, i.e., phagocytosed spirochetes (red).
